# Effect of diabetes mellitus on physical function in patients with osteoarthritis: a cross-sectional observational study

**DOI:** 10.3389/fendo.2025.1536341

**Published:** 2025-04-25

**Authors:** Zhiqiang Que, Wenbin Xu, Keyi Xiao, Cunbin Chen, Jianghao Shu, Yuxuan Huang, Dingqiang Chen, Gang Rui

**Affiliations:** ^1^ Department of Orthopedics, The First Affiliated Hospital of Xiamen University, School of Medicine, Xiamen University, Xiamen, China; ^2^ Department of Joint Surgery, Ningbo No. 6 Hospital, Ningbo, China; ^3^ The School of Clinical Medicine, Fujian Medical University, Fuzhou, China; ^4^ Xiamen Key Laboratory of Clinical Efficacy and Evidence Studies of Traditional Chinese Medicine, The First Affiliated Hospital of Xiamen University, School of Medicine, Xiamen University, Xiamen, China

**Keywords:** osteoarthritis, diabetes mellitus, physical function, National Health and Nutrition Examination Survey, cross sectional study

## Abstract

**Background:**

Osteoarthritis (OA) is a common chronic disease among the elderly, causing pain, functional limitations, and a decline in quality of life. Diabetes mellitus (DM), a prevalent metabolic disorder, has been proven to have an association with OA. However, the specific impact of DM on the physical function of OA patients remains lack of in-depth exploration. This study aims to investigate whether OA patients with DM (DMOA) experience more severe physical function limitations.

**Method:**

The study utilized National Health and Nutrition Examination Survey (NHANES) data from 1999-2018. Logistic regression models were used to analyze the association between DMOA and physical function limitations. Stratified analysis was applied to assess the stability of these results.

**Results:**

DMOA patients exhibited significantly worse physical function compared to those OA patiants who do not complicated with DM (non-DMOA), especially in high-intensity and frequent joint use activities like walking long distances (OR = 1.870, 95%CI[1.243,2.814], *P* = 0.003), crouching (OR = 1.417, 95%CI[1.116, 1.799], *P* = 0.005), and standing for long periods (OR = 1.423,95%CI[1.141,1.774], *P* = 0.002). Even after adjusting for demographics, socioeconomic and health factors, the association between DMOA and physical function impairment remained significant.

**Conclusion:**

This study revealed that the DMOA population has worse physical function than non-DMOA population, especially in high-intensity and frequent joint use activities. Managing DM in OA patients is crucial to improve their physical function and overall quality of life. The impact of DM should be considered in the selection of therapeutic agents and care for OA.

## 1 Background

Osteoarthritis (OA) is a whole joint disease characterized by the degeneration of articular cartilage, osteophyte formation, subchondral bone changes, and inflammation of the synovial membrane. These changes result in chronic pain, joint stiffness, and functional impairment, which severely impact the quality of life of individuals. OA is one of the most common chronic diseases after age 40 years and has become a major cause of disability in older adults, placing a substantial economic and health burden on society and individuals (1). As of 2020, approximately 595 million people globally were affected by OA, and this number is expected to increase with population aging (1). The deterioration of physical function is a key issue faced by OA patients, as impaired physical activity significantly reduces their quality of life and increases their reliance on healthcare services (2).

Traditionally, OA is considered a disease primarily caused by aging, mechanical joint stress, and trauma (3). However, growing attention has been given to additional factors influencing OA progression. In recent years, metabolic factors, for example obesity (4) and diabetes mellitus (DM) (5), have been revealed as important contributors to the development of OA. DM, a chronic metabolic disease affecting hundreds of millions of people worldwide, not only increases the risk of OA but may also worsen the severity of symptoms (6, 7). Studies, including meta-analyses (8) and long-term cohort investigations (9), have shown that patients with DM are more likely to develop OA, and their disease tends to be more severe, with greater pain and more pronounced structural destruction of the joints.

Although many researches (5, 8, 10) has established the association between DM and the increased risk of OA, there is a lack of in-depth exploration into the specific impact of DM on the physical function of DMOA patients. Since physical function impairment directly affects the quality of life of OA patients, determining whether DM exacerbates this impairment is of great importance for public health and clinical management. The purpose of this study is to use data from the National Health and Nutrition Examination Survey (NHANES) to investigate whether OA with comorbid DM (DMOA) exhibits more significant physical function decline. The findings from this research may provide valuable insights for more effective clinical interventions and care strategies aimed at improving the quality of life for DMOA patients, while also alleviating their disease burden.

## 2 Method

### 2.1 Data source and participants

The NHANES, conducted by the Centers for Disease Control and Prevention (CDC), serves to assess the health and nutritional status of adults and children in the United States (Https://www.cdc.gov/nchs/nhanes/, Accessed October 26, 2024). Since 1999, the survey has employed a nationally representative sample of approximately 5,000 individuals annually. The data for NHANES was gathered through interviews and physical examination, encompassing demographic, socioeconomic, dietary, and health-related factors. The examination component included medical, dental, and physiological measurements, as well as laboratory analyses. Data from NHANES are widely used in epidemiological research and inform the development of public health policies aimed at improving population health.

### 2.2 Definition of physical function limitations and other covariates

The physical function section (PFQ) provides self-reported data on functional limitations caused by long-term physical, mental, and emotional problems or illnesses. It can be used to assess an individual’s level of disability.

Questionnaire items that are highly correlated with joint function were selected to evaluate the physical function limitation of OA patients, including (1) Limitations keeping you from working; (2) Limited in amount of work you can do; (3) Need special equipment to walk; (4) Walking for a quarter mile difficulty; (5) Walking up ten steps difficulty; (6) Stooping crouching kneeling difficulty; (7) House chore difficulty; (8) Walking between rooms on same floor; (9) Standingup from armless chair difficulty; (10) Getting in and out of bed difficulty; (11) Standing for long periods difficulty. (1) and (2) these two questionnaire items, NHANES provide “Yes”, “No”, “Refused”, “Don’t know”, and “Missing” as the answer. In this study, we excluded the data for the latter three. The other nine questionnaire items record the results of “No difficulty”, “Some difficulty”, “Much difficulty”, “Unable to do”, “Do not do this activity”, “Refused”, “Don’t know”, and “Missing”. We reclassified “No difficulty” and “Some difficulty” as mild, and categorized “Much difficulty” and “Unable to do” combined as severe. The participants whose results were “Do not do this activity”, “Refused”, “Don’t know”, and “Missing” were excluded from this study.

Age, sex, ethnicity, marital status, poverty income ratio (PIR), education level, hypertension, smoking, alcohol use, stroke and coronary heart disease were selected as the covariates. Ethnicity was redivided as “Non-Hispanic White”, “Non-Hispanic Black”, “Mexican American” and “Other”; Marital status was classified as “Married or Living with partner”, “Never married”, and “Divorced or Widowed or Separated”. The PIR was classified as “0-1.3 RIP”, “> 1.3-3.5 RIP” and “> 3.5 RIP”; Education level was divided as “Less than 9th Grade”, “High School Grade or Equivalent”, and “College Graduate or above”; Smoking was divided as “Never”, “Former” and “Now”; Alcohol use was divided as “Never”, “Former”, “Mild”, “Moderate”, and “Heavy”. The BMI was classified as “Underweight”, “Normal”, “Overweight” and “Obese”.

### 2.3 Statistical analysis

All of the data analysis procedures were performed using the R (version 4.3.2) software. All data were weighted (wtint4yr or wtint2yr) before analysis. Continuous variables are presented as mean (SD) and categorical variables as number (%). The chi-square test was used to assess differences between two groups for categorical variables, and t-tests were used for continuous variables. Weighted multiple logistic regression models were used to assess the association of DMOA with physical function limitation. Stratified analyses were also performed to further assess the stability of these results. Results were considered to be significant when *P* value < 0.05.

## 3 Result

### 3.1 Participants characteristics

**Figure 1:**
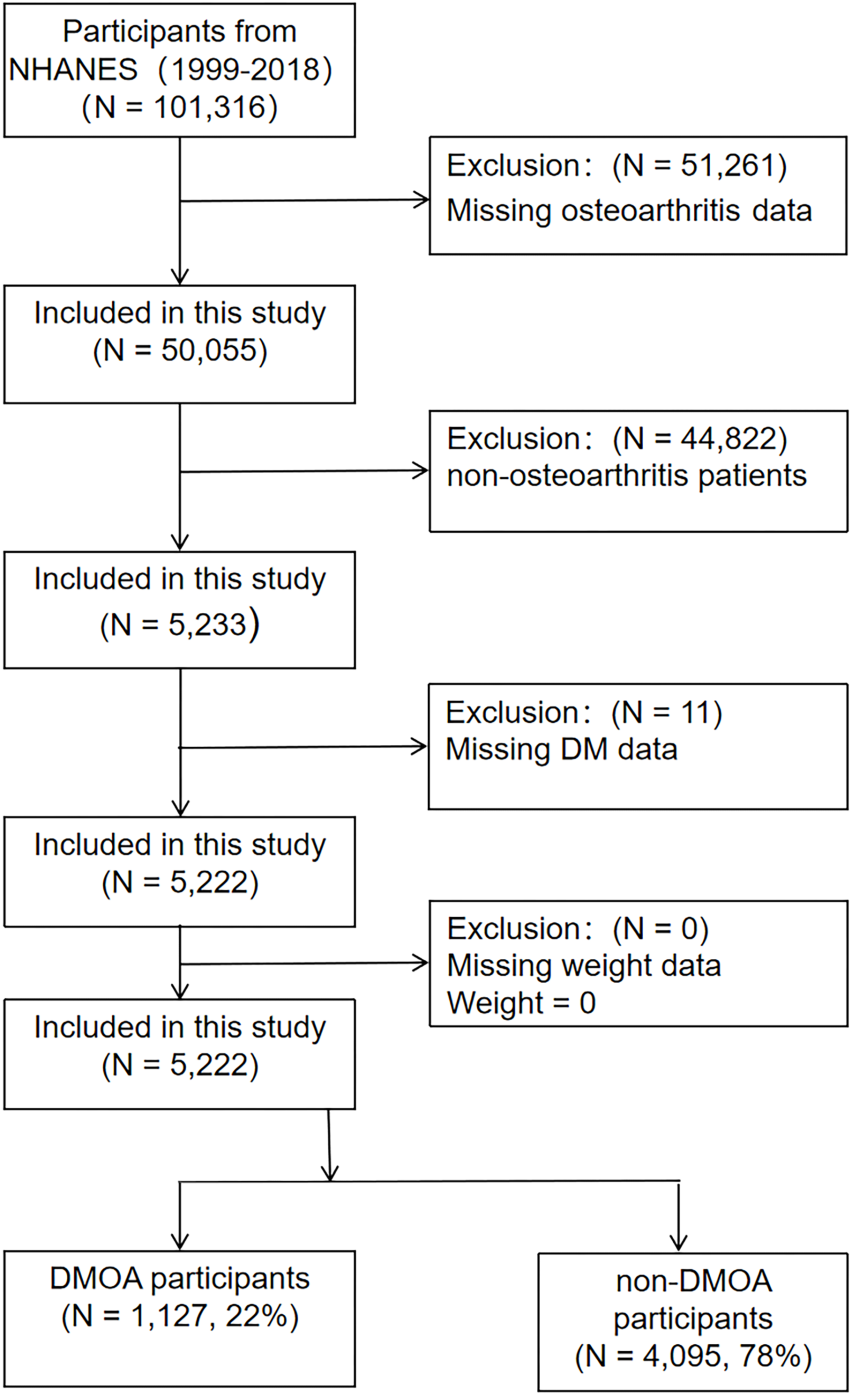
The participants selection flow.

A total of 10 cycles from 1999-2018 provided data on DM, OA, and physical function. Initially including 101,316 participants, after excluding 51,261 participants with missing OA data, 44,822 non-OA participants and 11 participants with missing DM information, a total of 5,222 OA patients were included in the study, including 1,127 DMOA patients and 4,095 non-DMOA patients. The participant selection process is presented in the **Figure 1**.

Weighted baseline characteristics of participants are shown in **Table 1**. The mean age of the DMOA group was 64.76 ± 0.52 years old, older than the non-DMOA group. The prevalence of DMOA was associated with race, education, income, BMI and lifestyle (smoking, alcohol consumption). Furthermore, the incidence of comorbidities was higher in the DMOA group than in the non-DMOA group.

**Table 1:** Baseline characteristics of the study participants in the NHANES.

Variable	Non-DMOA (N = 4,095)	DMOA (N = 1,127)	P value
Age	61.36 (0.25)	64.78 (0.52)	< 0.0001
Sex			0.33
Female	2650 (65.72)	707 (63.71)	
Male	1445 (34.28)	420 (36.29)	
Ethnicity			< 0.0001
Non-Hispanic White	2768 (84.93)	601 (76.12)	
Non-Hispanic Black	541 (5.71)	235 (9.77)	
Mexican American	320 (2.50)	120 (3.91)	
Other Race	466 (6.86)	171 (10.20)	
Marital status			0.11
Married or Living with partner	2360 (64.37)	599 (60.25)	
Never married	283 (7.16)	78 (6.66)	
Divorced or Widowed or Separated	1420 (28.47)	443 (33.10)	
Education level			< 0.0001
College Graduate or above	2306 (63.44)	511 (53.91)	
High School Grad or Equivalent	1447 (32.41)	463 (39.06)	
Less Than 9th Grade	338 (4.15)	149 (7.02)	
PIR			< 0.0001
0-1.3 PIR	904 (16.14)	340 (23.18)	
>1.3-3.5 PIR	1477 (35.96)	442 (43.93)	
>3.5 PIR	1349 (47.90)	242 (32.89)	
BMI			< 0.0001
Underweight	47 (1.02)	4 (0.27)	
Normal	901 (23.68)	91 (7.98)	
Overweight	1299 (34.20)	254 (21.08)	
Obese	1549 (41.10)	682 (70.68)	
Smoking			0.001
Never	1955 (47.94)	530 (45.75)	
Former	1446 (35.11)	450 (42.23)	
Now	691 (16.95)	146 (12.02)	
Alcohol use			< 0.0001
Never	474 (10.96)	184 (15.21)	
Former	737 (17.38)	296 (27.88)	
Mild	1456 (44.05)	311 (38.60)	
Moderate	496 (16.41)	90 (11.48)	
Heavy	363 (11.20)	54 (6.84)	
Hypertension			< 0.0001
No	1501 (41.58)	183 (18.59)	
Yes	2594 (58.42)	943 (81.41)	
Stroke			< 0.0001
No	3802 (94.54)	994 (88.45)	
Yes	283 (5.46)	132 (11.55)	
Coronary heart disease			< 0.0001
No	3750 (93.35)	917 (81.90)	
Yes	328 (6.65)	198 (18.10)	
Limitations keeping you from working			< 0.0001
No	3067 (78.81)	669 (65.72)	
Yes	1024 (21.19)	455 (34.28)	
Limited in amount of work you can do			< 0.0001
No	2020 (59.59)	398 (41.75)	
Yes	1606 (40.41)	649 (58.25)	
Walking for a quarter mile difficulty			< 0.0001
No difficulty	1660 (68.79)	322 (55.54)	
Some difficulty	560 (21.38)	141 (23.53)	
Much difficulty	182 (6.03)	60 (11.30)	
Unable to do	116 (3.21)	59 (8.88)	
Do not do this activity	26 (0.59)	5 (0.76)	
Walking up ten steps difficulty			< 0.001
No difficulty	1881 (77.22)	378 (67.14)	
Some difficulty	461 (16.72)	132 (21.87)	
Much difficulty	124 (4.17)	42 (6.72)	
Unable to do	62 (1.46)	30 (3.27)	
Do not do this activity	17 (0.44)	5 (1.00)	
Stooping crouching kneeling difficulty			< 0.0001
No difficulty	1104 (34.01)	215 (21.87)	
Some difficulty	1185 (35.74)	322 (30.45)	
Much difficulty	680 (18.82)	264 (24.72)	
Unable to do	399 (9.38)	215 (19.47)	
Do not do this activity	77 (2.05)	34 (3.49)	
House chore difficulty			< 0.0001
No difficulty	2040 (63.52)	457 (45.53)	
Some difficulty	828 (22.63)	298 (29.37)	
Much difficulty	283 (8.36)	130 (12.61)	
Unable to do	182 (3.32)	101 (6.80)	
Do not do this activity	112 (2.17)	64 (5.68)	
Walking between rooms on same floor			< 0.0001
No difficulty	2850 (86.54)	738 (73.29)	
Some difficulty	398 (9.59)	203 (18.38)	
Much difficulty	120 (2.40)	60 (4.33)	
Unable to do	64 (1.24)	43 (3.41)	
Do not do this activity	13 (0.23)	6 (0.58)	
Standing up from armless chair difficulty			< 0.0001
No difficulty	2172 (66.82)	503 (52.13)	
Some difficulty	886 (24.49)	344 (31.09)	
Much difficulty	254 (6.24)	122 (10.49)	
Unable to do	125 (2.28)	73 (5.43)	
Do not do this activity	9 (0.18)	8 (0.86)	
Getting in and out of bed difficulty			< 0.0001
No difficulty	2517 (76.32)	628 (64.15)	
Some difficulty	716 (19.08)	316 (26.12)	
Much difficulty	176 (3.87)	81 (6.93)	
Unable to do	32 (0.59)	16 (1.34)	
Do not do this activity	5 (0.14)	9 (1.46)	
Standing for long periods difficulty			< 0.0001
No difficulty	1309 (41.21)	285 (28.90)	
Some difficulty	900 (27.34)	238 (22.50)	
Much difficulty	554 (15.93)	186 (17.06)	
Unable to do	603 (13.57)	302 (27.79)	
Do not do this activity	79 (1.96)	38 (3.74)	

PIR, poverty income ratio; DMOA, osteoarthritis patients with diabetes; Non-DMOA, osteoarthritis patients without diabetes. Continuous variables are presented as mean (SD) and categorical variables as number (%).

### 3.2 Association between DMOA and physical function

Three logistic regression models were constructed and used to assess the relationship between DMOA and physical function limitation. The crude model did not adjust for any covariates; Model 1 adjusted for age, sex, ethnicity, marital status, PIR and education level; Model 2 adjusted for age, sex, ethnicity, marital status, PIR, education level, hypertension, diabetes, smoking, alcohol use, hypertension, stroke, coronary heart disease. In the crude model, DMOA showed significant correlation with all of the physical function metrics, and the physical function of the DMOA populations performed worse (*P* < 0.05). After fully adjusting for all of the covariates, Only “Limited in amount of work you can do” (OR = 1.622, 95%CI[1.250,2.104], *P* < 0.001), “Need special equipment to walk” (OR = 1.712, 95%CI[1.312,2.234], *P* < 0.001), “Walking for a quarter mile difficulty” (OR = 1.870, 95%CI[1.243,2.814], *P* = 0.003), “Stooping crouching kneeling difficulty” (OR = 1.417, 95%CI[1.116, 1.799], *P* = 0.005), “Standing for long periods difficulty” (OR = 1.423,95%CI[1.141,1.774], *P* = 0.002) still showed a significant correlation (*P* < 0.05), but the correlation of other physical function evaluation indicators is no longer significant (*P* > 0.05). Detailed information is displayed in **Table 2**.

**Table 2:** Multivariate logistic regression analysis between DMOA and physical function.

Physical function index	Number of participants			Crude model		Model 1		Model 2	
	Total	DMOA	non- DMOA	OR (95%CI)	P value	OR (95%CI)	P value	OR (95%CI)	P value
Limitations keeping you from working	5215	1124	4091	1.939 (1.622,2.319)	<0.0001	1.812 (1.488,2.207)	<0.0001	1.315 (1.042,1.659)	0.022
Limited in amount of work you can do	4673	1047	3626	2.058 (1.645,2.575)	<0.0001	2.003 (1.588,2.527)	<0.0001	1.622 (1.250,2.104)	<0.001
Need special equipment to walk	4678	1048	3630	2.725 (2.204,3.368)	<0.0001	2.380 (1.862,3.043)	<0.0001	1.712 (1.312,2.234)	<0.001
Walking for a quarter mile difficulty	3100	582	2518	2.490 (1.749,3.545)	<0.0001	2.357 (1.518,3.660)	<0.001	1.870 (1.243,2.814)	0.003
Walking up ten steps difficulty	3110	582	2528	1.873 (1.240,2.827)	0.003	1.457 (0.951,2.232)	0.083	1.007 (0.614, 1.652)	0.979
Stooping crouching kneeling difficulty	4384	1016	3368	2.089 (1.725,2.531)	<0.0001	1.945 (1.580,2.395)	<0.0001	1.417 (1.116, 1.799)	0.005
House chore difficulty	4319	986	3333	1.912 (1.478,2.475)	<0.0001	1.781 (1.321,2.403)	<0.001	1.453 (0.993, 2.127)	0.054
Walking between rooms on same floor	4476	1044	3432	2.234 (1.579,3.161)	<0.0001	1.860 (1.225,2.824)	0.004	1.438 (0.885, 2.334)	0.141
Standing up from armless chair difficulty	4479	1042	3437	2.051 (1.520,2.769)	<0.0001	1.719 (1.249,2.366)	0.001	1.396 (0.956, 2.037)	0.084
Getting in and out of bed difficulty	4482	1041	3441	1.963 (1.368,2.816)	<0.001	1.544 (1.019,2.340)	0.041	1.644 (0.996, 2.712)	0.052
Standing for long periods difficulty	4377	1011	3366	2.028 (1.691,2.433)	<0.0001	1.847 (1.504,2.267)	<0.0001	1.423 (1.141,1.774)	0.002

Crude model: Without any adjustment.

Model 1: age, sex, ethnicity, marital status, PIR, education level.

Model 2: age, sex, ethnicity, marital status, PIR, education level, BMI, smoking, alcohol use, hypertension, stroke, coronary heart disease.

PIR, poverty income ratio; DMOA, osteoarthritis patients with diabetes; Non-DMOA, osteoarthritis patients without diabetes; OR, odds ratio; 95% CI, 95% confidence interval.

### 3.3 Stratified analysis between DMOA and physical function

**Figure 2:**
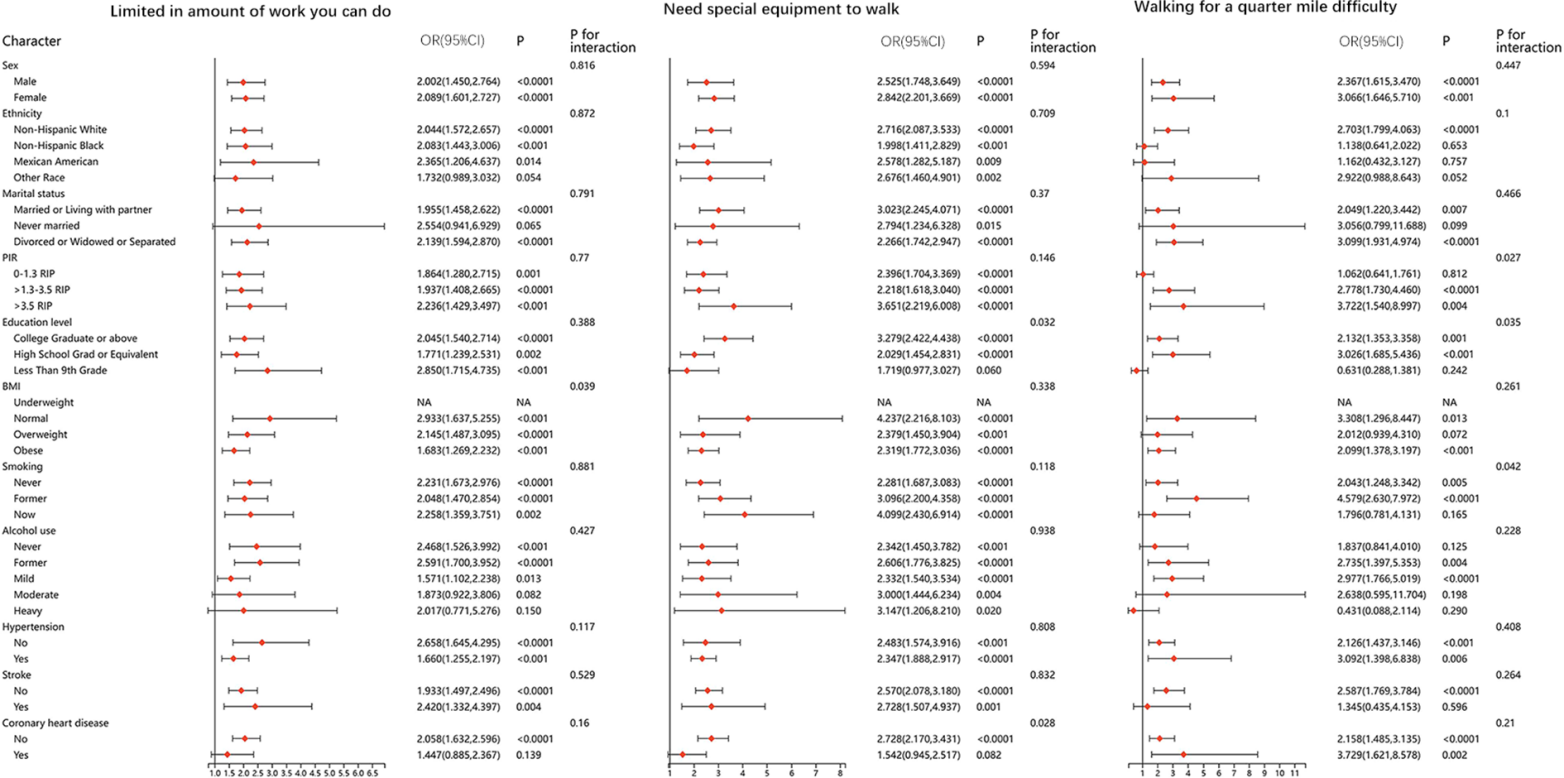
Stratified analysis between DMOA and physical function limitation.

**Figure 3:**
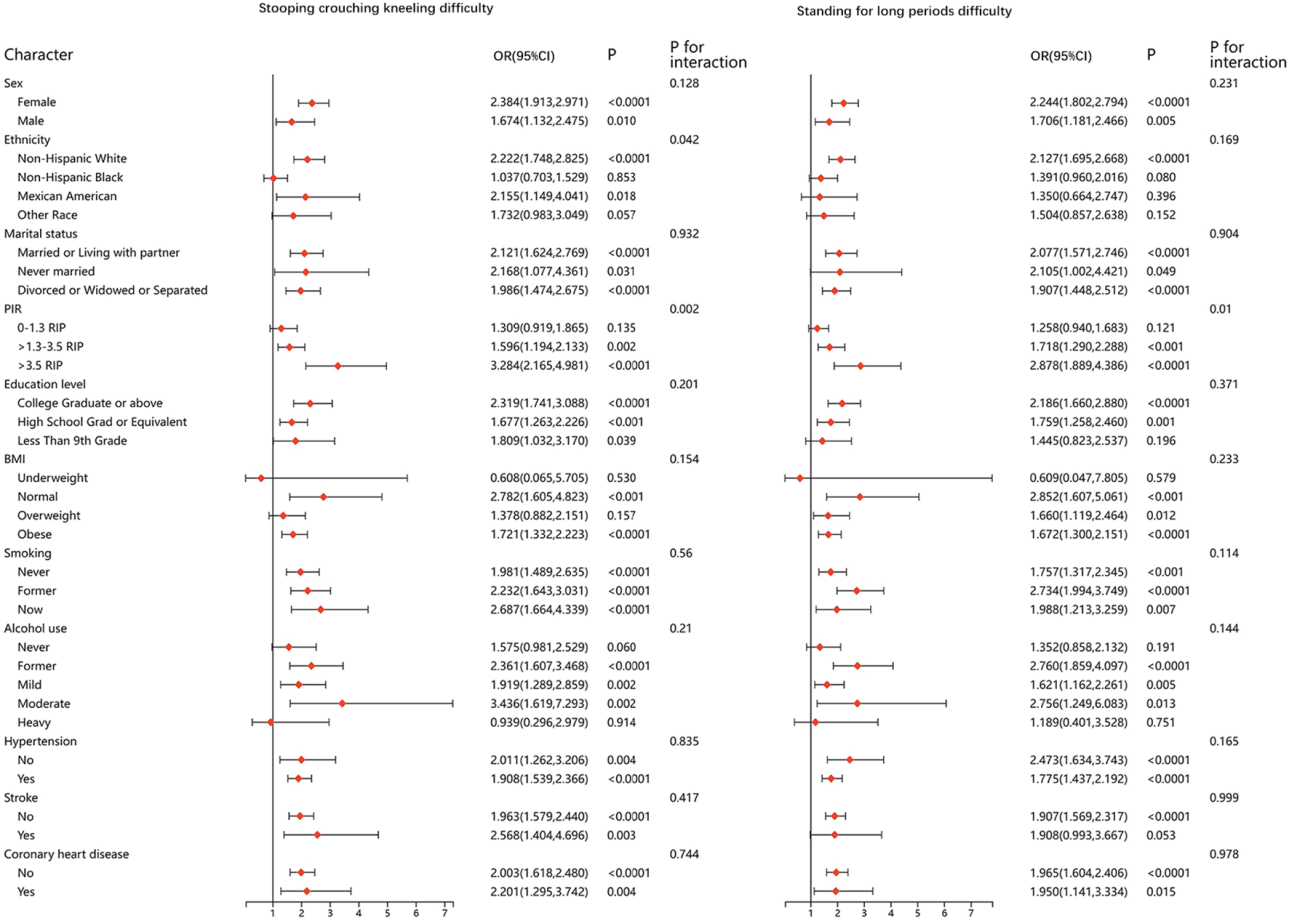
Stratified analysis between DMOA and physical function limitation.

We performed stratified analyses for all of the physical function metrics. The results were stable in most of the stratified population, and no significant differences were found. Results of stratified analyses of the five questionnaire items that remained significantly associated after adjusting for all covariates are presented in **Figures 2** and **3**. Results for the remaining questionnaire items are presented in **Supplementary Figures 1** and **2**.

## 4 Discussion

This study evaluated the differences in physical function between DMOA and non-DMOA patients, based on the NHANES data. The results revealed that patients with DMOA had significantly worse physical function than those with non-DMOA. These findings support the view that DM is an important factor for the progression of OA, as proposed in previous studies (9, 11, 12).

In this study, after adjusting for demographic and socioeconomic factors such as age, gender, race, marital status, education level and PIR, the difference of physical function between DMOA and non-DMOA is still significant. However, some associations became non-significant with further adjustment for lifestyle factors such as BMI, smoking and drinking habits, as well as other chronic diseases such as hypertension, stroke, and coronary heart disease. After adjusting for all the relevant covariates, “Walking for a quarter mile difficulty”, “Stooping crouching kneeling difficulty”, “Standing for long periods difficulty”, etc., the DMOA group still showed significantly worse performance than the non-DMOA group. The indicators of physical function that become insignificant are mostly those activities with relatively mild exercise intensity or less range of joint motion, such as “Walking up ten steps difficulty”, “House chore difficulty”, “Walking between rooms on same floor”, “Standing up from armless chair difficulty”, and “Getting in and out of bed difficulty” etc.

The direct association of DMOA with physical function limitation was diminished after considering more complex health factors. This phenomenon suggests that although the influence of DM in patients with OA cannot be ignored, other lifestyle and chronic diseases also have important effects on physical function in OA patients. The prevalence, progression, and severity of OA can be influenced by several factors, including sex, age, obesity, lifestyle, diet, genetics, and comorbidities (13). Age is one of the main risk factors for OA, and the prevalence of OA increases with increasing age. According to the Global Burden of Disease Study (GBD), the global prevalence of OA in people over 70 years is about 15% higher than in those aged 50-69 years (1). Gender is also an important factor affecting the prevalence of OA. Studies (14, 15) have shown that women are more likely to develop OA, especially after menopause. Globally, women account for 60% of osteoarthritis cases (16). This is associated with a range of biological factors (e.g., hormone, neurological, immune regulation, and genetic factors), differences in joint anatomy, muscle strength, and ligament relaxation, and lifestyle factors between men and women (17, 18). Physical activity and occupation have important effects on the progression of OA. Individuals who are engaged in high intensity sport or repetitive joint movements for a long time (such as athlete, construction workers or agricultural workers) are at higher risk of developing OA (19–21). In terms of diet, anti-inflammatory diets (such as foods rich in Omega-3 fatty acids and antioxidants) may help reduce the inflammatory response in OA (22). In addition, an adequate intake of calcium and vitamin D is also crucial for maintaining joint health (23).

The reasons for the absence of a significant difference between DMOA and non-DMOA groups in mild exercise intensity or less range of joint motion may be as follow. Low-intensity exercise can be easily completed both in patients with early OA and in patients with advanced OA. Even if DM can accelerate the progression of OA and lead to severe symptoms, it is not easy to show in these mild activities. A cross-sectional study by Fujita et al (12) showed that Knee OA patients with DM had significantly lower physical activity levels than those without DM. Their conclusion is consistent with our study, and the participants included in this study were only moderate-severe OA patients, excluding mild OA patients. Another longitudinal study (24) showed that antidiabetic medication for diabetes were only able to reduce the progression of knee OA but had no effect on the incidence of knee OA. From this point, we can also speculate that the effect of diabetes on early OA may not be significant.

The significant negative impact of DM on physical function in OA patients during high exercise intensity or frequent joint use can be explaned as follows. Diabetic patients are often do not adequately engage in physical activity, and the lack of physical activity is also one of the important risk factors for the occurrence and development of OA. This may be one of the mediators of the association of diabetes with OA. Furthermore, recent studies have gradually revealed the mechanisms of multiple interactions between DM and OA. One of the main pathological features of DM is long-term hyperglycemia, which triggers a series of physiological changes that aggravate joint inflammation and degenerative lesions. In diabetic patients, long-term hyperglycemia leads to the accumulation of glycation end products (AGEs), which activate the proinflammatory signaling pathway by binding to their receptor (RAGE), and then destroy the structure and function of articular cartilage and synovial membrane (25–27). The accumulation of AGEs also leads to the degeneration of cartilage matrix proteins, weakening the repair ability of cartilage tissue, thus accelerating the development of OA (28). Moreover, AGEs can also enhance the oxidative stress response and further aggravate joint damage (29). This mechanism explains why the joint degenerative change is severe in diabetic patients. DM is usually accompanied by a systemic chronic low-grade inflammatory response, with proinflammatory cytokines such as TNF-α and IL-6 having elevated levels in DM patients (30). These inflammatory factors can accelerate the degeneration of articular cartilage and play an important role in the pathological process of OA. Previous studies have shown that chronic inflammation in diabetic patients may aggravate the OA condition via a systemic inflammatory response, leading to more severe joint pain and functional impairment (11, 13, 31). DM usually exists with metabolic disorders such as obesity and hypertension, all of which further worsen joint inflammation by increasing mechanical loading on the joint or through pro-inflammatory mechanisms. Obesity increases the mechanical pressure on the joints, and pro-inflammatory factors such as leptin produced by the combination of obesity and diabetes also play an important role in the progression of OA (32, 33). The multiple effects of the metabolic syndrome may be one of the reasons why more limited physical functions in DMOA patients.

The presence of DM significantly impacts the choice of medication and care strategies for OA patients (5). Studies have shown that the safety of some commonly used OA medications in DM patients carries potential risks. For example, although acetaminophen is widely used for OA pain management, its hepatotoxicity raises concerns, however, T2DM patients often suffer from non-alcoholic fatty liver disease (NAFLD) and more severe steatohepatitis (NASH) (34, 35). Besides, animal research has also suggested that the toxicity of acetaminophen is exacerbated in the condition of presence of DM (36). Therefore, its safety in DMOA patients remains a matter of concern. Non-steroidal anti-inflammatory drugs (NSAIDs) are effective in relieving pain and inflammation but may increase the risk of hospitalization in DM patients (37). Additionally, sodium-glucose co-transporter-2 (SGLT2) inhibitors, a commonly used class of antidiabetic drugs, may impair kidney function and exacerbate the adverse effects of NSAIDs. Therefore, caution is advised when prescribing NSAIDs to OA patients taking SGLT2 inhibitors (38). On the other hand, intra-articular corticosteroid injections can provide short-term relief of OA symptoms but may cause significant elevations in blood glucose levels in DM patients, thereby increasing the risk of hyperglycemia-related complications. Consequently, it is recommended that blood glucose levels be closely monitored for 24-48 hours post-injection, with adjustments to antidiabetic treatment made as needed (39). Overall, treatment strategies for patients with coexisting DM and OA should not only focus on pain relief and inflammation control but also consider metabolic safety to optimize individualized treatment plans, minimize drug-related adverse events, and improve long-term patient outcomes.

In summary, the results of this study further support the idea that there is a complex interaction between DM and OA. DM is not only a risk factor for OA, but also has a significant association with physical function limitations in patients. Therefore, in addition to routine joint protection and pain management in DMOA patients, the management of diabetes should be regarded as one of the important therapeutic goals, and the impact of DM should be considered in therapeutic drug selection and care.

## 5 Advantage and and limitation

This study has several advantages. First, large-scale, nationally representative data of NHANES were used to ensure the broad applicability of the results. Second, the multivariable adjustment reduced the interference of confounders and made the study results more reliable. However, some limitations also can’t be ignored. First, the cross-sectional nature of NHANES data prevents us from establishing a causal association between DM and OA-related functional decline. It remains unclear whether DM exacerbates functional impairment or whether reduced physical function contributes to DM in OA patients. Future longitudinal and interventional studies are needed to clarify this relationship. Second, this study primarily relied on self-reported diagnoses of DM and OA, which may introduce recall bias and limit our ability to assess disease duration, severity, and treatment effects. Additionally, NHANES lacks standardized radiographic assessments, such as the Kellgren-Lawrence grading system, which would provide a more objective evaluation of OA severity. Third, while we adjusted for multiple confounders in our regression models, several important factors—such as pain intensity, arthritis medication use, rehabilitation therapy, and occupational workload—were not consistently available in NHANES and thus could not be included in our analysis. Lastly, our functional assessment relied on self-reported physical activity limitations, which may not accurately distinguish between subjective willingness and objective ability. Additionally, the exclusion of individuals who refused to answer or reported not engaging in certain activities may introduce selection bias. Future studies should incorporate objective physical function measurements, such as gait speed, or accelerometer-based activity tracking, to enhance data reliability.

## 6 Conclusion

This study revealed that the DMOA population shows worse physical function than non-DMOA population, and this difference was more obvious in activities with greater activity intensity or more frequent and wider joint activity. Managing DM in OA patients is crucial to improve their physical function and overall quality of life. The impact of DM should be considered in the selection of therapeutic agents and care for OA.

## Data Availability

Publicly available datasets were analyzed in this study. This data can be found here: http://www.cdc.gov/nchs/nhanes/.
