# Supplementary material for: Effect of diabetes mellitus on physical function in patients with osteoarthritis: a cross-sectional observational study
Source: Front Endocrinol (Lausanne). 2025 Apr 25;16:1536341. doi: 10.3389/fendo.2025.1536341 (PMC12061731; doi:10.3389/fendo.2025.1536341)

Supplementary Figure 1. Stratified analysis between DMOA and physical function limitation.

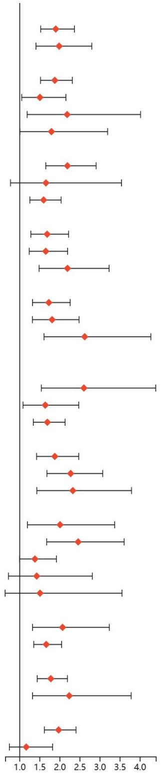

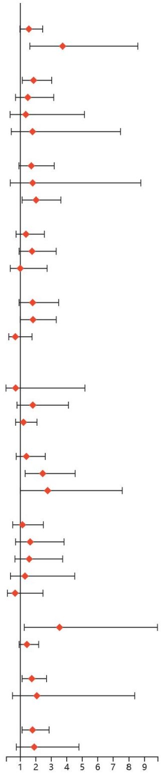

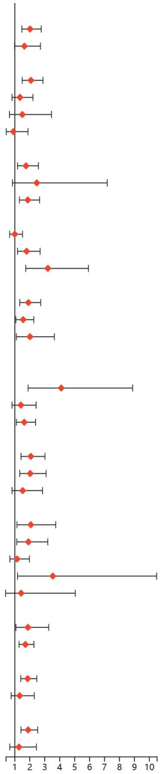

Character	Limitations keeping you from working	P	P for interaction	Walking up ten steps difficulty	P	P for interaction	House chore difficulty	P	P for interaction
Sex			0.831			0.068			0.5
Female	1.908(1.534,2.373)	<0.0001		1.576(1.003,2.477)	0.049		2.047(1.497,2.800)	<0.0001	
Male	1.993(1.415,2.807)	<0.001		3.761(1.645,8.595)	0.002		1.671(1.017,2.745)	0.043	
Ethnicity			0.773			0.955			0.075
Non-Hispanic White	1.884(1.528,2.322)	<0.0001		1.881(1.157,3.059)	0.011		2.106(1.521,2.917)	<0.0001	
Non-Hispanic Black	1.513(1.059,2.161)	0.023		1.511(0.717,3.186)	0.272		1.370(0.838,2.240)	0.206	
Mexican American	2.192(1.195,4.022)	0.013		1.381(0.368,5.175)	0.619		1.528(0.671,3.480)	0.302	
Other Race	1.801(1.014,3.201)	0.045		1.818(0.441,7.505)	0.401		0.931(0.453,1.911)	0.843	
Marital status			0.297			0.921			0.806
Married or Living with partner	2.199(1.659,2.914)	<0.0001		1.737(0.937,3.219)	0.079		1.777(1.210,2.609)	0.004	
Never married	1.663(0.780,3.547)	0.184		1.826(0.379,8.789)	0.439		2.498(0.865,7.211)	0.089	
Divorced or Widowed or Separated	1.605(1.262,2.041)	<0.001		2.046(1.147,3.650)	0.016		1.898(1.340,2.687)	<0.001	
PIR			0.406			0.578			0.009
0-1.3 RIP	1.693(1.287,2.228)	<0.001		1.392(0.751,2.580)	0.290		1.026(0.682,1.545)	0.901	
>1.3-3.5 RIP	1.659(1.248,2.205)	<0.001		1.784(0.951,3.346)	0.071		1.816(1.211,2.723)	0.004	
>3.5 RIP	2.200(1.493,3.239)	<0.0001		1.022(0.379,2.760)	0.965		3.237(1.759,5.958)	<0.001	
Education level			0.475			0.263			0.6
College Graduate or above	1.736(1.329,2.269)	<0.0001		1.824(0.949,3.507)	0.071		1.938(1.361,2.759)	<0.001	
High School Grad or Equivalent	1.817(1.326,2.489)	<0.001		1.848(1.020,3.350)	0.043		1.587(1.096,2.297)	0.015	
Less Than 9th Grade	2.629(1.615,4.279)	<0.001		0.703(0.277,1.785)	0.450		2.041(1.134,3.675)	0.018	
BMI			0.017			0.606			0.082
Underweight	NA	NA		NA	NA		NA	NA	
Normal	2.610(1.549,4.400)	<0.001		0.725(0.101,5.204)	0.747		4.136(1.918,8.915)	<0.001	
Overweight	1.647(1.092,2.482)	0.018		1.834(0.812,4.139)	0.143		1.432(0.837,2.450)	0.188	
Obese	1.699(1.348,2.142)	<0.0001		1.232(0.721,2.104)	0.442		1.658(1.136,2.419)	0.009	
Smoking			0.587			0.311			0.69
Never	1.884(1.430,2.482)	<0.0001		1.418(0.762,2.642)	0.268		2.095(1.442,3.043)	<0.001	
Former	2.280(1.688,3.080)	<0.0001		2.476(1.341,4.572)	0.004		2.058(1.356,3.123)	<0.001	
Now	2.335(1.437,3.797)	<0.001		2.793(1.024,7.618)	0.045		1.554(0.839,2.879)	0.159	
Alcohol use			0.251			0.846			0.279
Never	2.015(1.202,3.378)	0.008		1.164(0.539,2.516)	0.695		2.101(1.172,3.765)	0.013	
Former	2.467(1.683,3.614)	<0.0001		1.664(0.719,3.852)	0.231		1.940(1.163,3.235)	0.012	
Mild	1.390(1.005,1.924)	0.047		1.601(0.681,3.764)	0.278		1.180(0.695,2.005)	0.537	
Moderate	1.433(0.729,2.819)	0.294		1.331(0.390,4.544)	0.644		3.569(1.211,10.521)	0.022	
Heavy	1.516(0.646,3.560)	0.334		0.690(0.191,2.492)	0.562		1.450(0.414,5.075)	0.555	
Hypertension			0.411			0.117			0.757
No	2.077(1.330,3.244)	0.001		3.560(1.285,9.865)	0.015		1.906(1.102,3.298)	0.021	
Yes	1.671(1.358,2.055)	<0.0001		1.452(0.953,2.213)	0.082		1.740(1.313,2.307)	<0.001	
Stroke			0.448			0.806			0.256
No	1.784(1.446,2.200)	<0.0001		1.766(1.146,2.719)	0.010		1.889(1.428,2.498)	<0.0001	
Yes	2.244(1.329,3.788)	0.003		2.094(0.522,8.396)	0.286		1.351(0.784,2.330)	0.274	
Coronary heart disease			0.037			0.903			0.285
No	1.979(1.624,2.413)	<0.0001		1.815(1.142,2.885)	0.012		1.918(1.438,2.560)	<0.0001	
Yes	1.172(0.751,1.829)	0.480		1.925(0.769,4.816)	0.158		1.303(0.688,2.470)	0.412	

Supplementary Figure 2. Stratified analysis between DMOA and physical function limitation.

Character	Walking between rooms on same floor	P	P for interaction	Standing up from armless chair difficulty	P	P for interaction	Getting in and out of bed difficulty	P	P for interaction
Sex			0.534			0.555			0.239
Female	2.422(1.726,3.400)	<0.0001		1.938(1.411,2.662)	<0.0001		2.363(1.608,3.474)	<0.0001	
Male	1.879(0.861,4.102)	0.112		2.376(1.270,4.448)	0.007		1.421(0.667,3.029)	0.360	
Ethnicity			0.935			0.462			0.203
Non-Hispanic White	2.167(1.375,3.417)	0.001		2.084(1.468,2.957)	<0.0001		2.195(1.446,3.330)	<0.001	
Non-Hispanic Black	1.636(0.834,3.209)	0.149		1.217(0.696,2.128)	0.485		0.780(0.373,1.630)	0.503	
Mexican American	1.711(0.634,4.612)	0.279		2.415(1.180,4.943)	0.017		1.218(0.544,2.725)	0.622	
Other Race	2.143(0.619,7.425)	0.225		1.652(0.670,4.076)	0.271		1.396(0.425,4.588)	0.577	
Marital status			0.308			0.66			0.5
Married or Living with partner	2.856(1.594,5.117)	<0.001		2.236(1.475,3.389)	<0.001		2.266(1.323,3.882)	0.003	
Never married	1.581(0.412,6.075)	0.496		1.830(0.629,5.324)	0.260		1.223(0.383,3.909)	0.729	
Divorced or Widowed or Separated	1.758(1.147,2.694)	0.010		1.774(1.231,2.555)	0.002		1.677(1.025,2.744)	0.040	
PIR			0.092			0.455			0.115
0-1.3 RIP	1.432(0.836,2.453)	0.189		1.535(1.021,2.307)	0.040		1.137(0.714,1.811)	0.587	
>1.3-3.5 RIP	1.827(0.939,3.555)	0.075		1.705(1.073,2.709)	0.024		1.520(0.812,2.845)	0.189	
>3.5 RIP	4.081(1.956,8.516)	<0.001		2.479(1.276,4.816)	0.008		3.577(1.347,9.499)	0.011	
Education level			0.392			0.422			0.652
College Graduate or above	2.613(1.508,4.526)	<0.001		1.896(1.209,2.974)	0.006		2.165(1.139,4.113)	0.019	
High School Grad or Equivalent	1.729(1.031,2.901)	0.038		2.231(1.406,3.540)	<0.001		1.686(0.938,3.031)	0.081	
Less Than 9th Grade	1.706(0.887,3.278)	0.108		1.301(0.743,2.279)	0.352		1.392(0.693,2.794)	0.347	
BMI			0.034			0.361			0.121
Underweight	NA	NA		NA	NA		NA	NA	
Normal	7.309(2.249,23.746)	0.001		2.682(1.144,6.288)	0.024		5.047(1.780,14.312)	0.003	
Overweight	1.191(0.461,3.075)	0.717		2.239(1.220,4.110)	0.010		1.073(0.407,2.825)	0.886	
Obese	1.760(1.110,2.792)	0.017		1.598(1.092,2.338)	0.016		1.998(1.163,3.431)	0.013	
Smoking			0.186			0.089			0.041
Never	1.898(1.240,2.906)	0.003		1.652(1.177,2.318)	0.004		1.675(1.069,2.626)	0.025	
Former	3.263(1.849,5.758)	<0.0001		3.050(1.912,4.865)	<0.0001		3.455(1.715,6.960)	<0.001	
Now	1.424(0.502,4.037)	0.502		1.690(0.783,3.647)	0.179		0.972(0.392,2.410)	0.951	
Alcohol use			0.183			0.269			0.418
Never	2.151(1.050,4.409)	0.037		1.096(0.628,1.914)	0.744		1.404(0.728,2.709)	0.307	
Former	1.557(0.762,3.182)	0.222		2.016(1.138,3.573)	0.017		1.529(0.819,2.854)	0.181	
Mild	2.538(1.112,5.790)	0.027		1.684(0.945,3.002)	0.077		1.747(0.775,3.940)	0.177	
Moderate	0.377(0.048,2.940)	0.347		3.853(1.358,10.933)	0.012		2.866(0.656,12.520)	0.159	
Heavy	5.257(1.415,19.534)	0.014		3.661(0.926,14.483)	0.064		4.670(1.299,16.783)	0.019	
Hypertension			0.37			0.265			0.56
No	2.952(1.365,6.385)	0.006		2.615(1.385,4.936)	0.003		2.438(1.124,5.290)	0.024	
Yes	2.044(1.430,2.921)	<0.001		1.793(1.309,2.457)	<0.001		1.927(1.327,2.799)	<0.001	
Stroke			0.482			0.451			0.996
No	1.819(1.251,2.644)	0.002		1.970(1.416,2.740)	<0.0001		1.754(1.230,2.501)	0.002	
Yes	2.317(1.253,4.287)	0.008		1.549(0.871,2.756)	0.134		1.757(0.818,3.775)	0.146	
Coronary heart disease			0.644			0.439			0.085
No	2.223(1.548,3.193)	<0.0001		2.090(1.493,2.925)	<0.0001		2.174(1.504,3.142)	<0.0001	
Yes	1.851(0.830,4.130)	0.131		1.595(0.842,3.022)	0.150		1.064(0.457,2.475)	0.884	

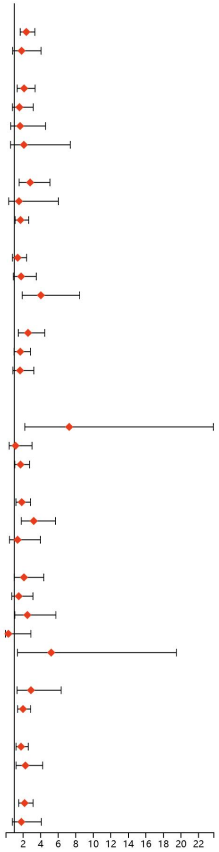

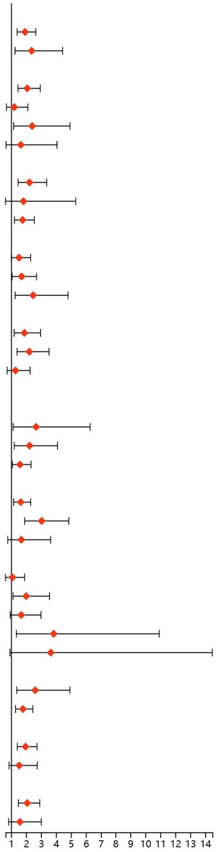

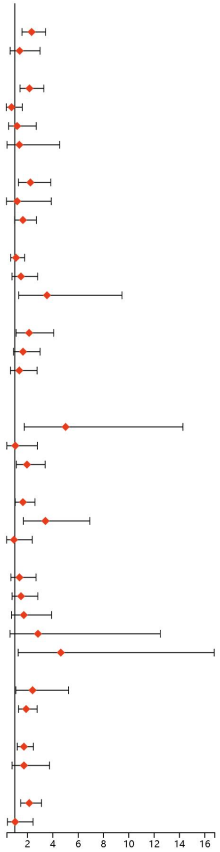

Supplement: Supplementary file 1 [file Image1.pdf]
